# Shoulder External Rotation Force Steadiness and Closed-Chain Reach Performance in Healthy Young Adults: An Exploratory Study

**DOI:** 10.7759/cureus.117704

**Published:** 2026-10-09

**Authors:** Atsushi Ueda, Yasuhiro Mitani, Hitoshi Koda, Toshimitsu Omine, Mizuki Nakamura

**Affiliations:** 1 Department of Rehabilitation Sciences, Faculty of Allied Health Sciences, Kansai University of Welfare Sciences, Kashiwara, JPN; 2 Faculty of Arts and Sciences, Kyoto Institute of Technology, Kyoto, JPN

**Keywords:** closed-chain upper-extremity performance, force steadiness, force variability, handheld dynamometer, shoulder external rotation, upper-extremity reach performance

## Abstract

Introduction

Maximal shoulder external rotation strength may not fully reflect force regulation during sustained submaximal contractions. This exploratory study examined whether shoulder external rotation force steadiness, quantified by force variability during 20% and 40% maximal voluntary contraction (MVC) tasks, was associated with normalized medial, superolateral, and inferolateral reach distances on the upper-extremity Star Excursion Balance Test (UE-SEBT) in healthy young adults. MVC peak force was also examined to compare maximal force-generating capacity with submaximal force regulation.

Materials and methods

Twenty-six healthy young adults participated in this cross-sectional study. Shoulder external rotation MVC peak force and force steadiness at 20% and 40%MVC were assessed using a pull-type handheld dynamometer (Mobie Z; Sakai Medical, Japan). Force variability was quantified using the standard deviation (SD) and coefficient of variation (CV) from the five-second window with the lowest SD during a 15-second submaximal task. Medial, superolateral, and inferolateral UE-SEBT reach distances were normalized to upper-limb length. Raw correlation p-values were adjusted using the Benjamini-Hochberg false discovery rate procedure. Exploratory body-mass-adjusted partial correlations were calculated for selected variables.

Results

Before correction for multiple comparisons, 20%MVC SD correlated negatively with medial reach (ρ=-0.503, raw p=0.009) and superolateral reach (ρ=-0.528, raw p=0.006). The 20%MVC CV correlated negatively with medial reach (r=-0.421, raw p=0.032) and MVC peak force with superolateral reach (r=-0.484, raw p=0.012). None remained statistically significant after Benjamini-Hochberg false discovery rate correction. Exploratory body-mass adjustment attenuated the MVC peak force correlation but minimally changed the 20%MVC force variability correlations. Force variability during the 40%MVC task showed no consistent associations with UE-SEBT reach distances.

Conclusions

In healthy young adults, selected shoulder external rotation force variability indices showed moderate negative correlations with UE-SEBT reach distances before correction for multiple comparisons. None remained statistically significant after false discovery rate correction. These findings are exploratory and hypothesis-generating rather than confirmatory.

## Introduction

The rotator cuff muscles contribute to humeral head control during upper-extremity movement [1,2]. The posterior rotator cuff muscles, including the infraspinatus and teres minor, contribute to shoulder external rotation and control during loaded or sport-specific tasks [3,4]. Their dysfunction may contribute to shoulder instability, rotator cuff-related shoulder pain, and functional deficits in overhead athletes [2,5,6]. Assessing shoulder external rotation function is therefore clinically relevant in sports rehabilitation and shoulder evaluation.

Shoulder external rotation strength is commonly reduced in individuals with shoulder disorders and frequently assessed in clinical and sports rehabilitation [1,2]. Shoulder external rotation function is often assessed using maximal strength measures. Maximal strength measures indicate force-generating capacity but may not fully characterize force regulation during sustained submaximal contractions [7]. Daily activities, upper extremity weight-bearing tasks, and sport-related movements often require fine submaximal force regulation, postural control, and coordinated upper-extremity movement rather than maximal force production.

Force steadiness is the ability to maintain a target submaximal force with minimal fluctuation over time [7]. It is commonly quantified using variability indices during sustained submaximal contraction [7,8]. The standard deviation (SD) reflects absolute force variability, whereas the coefficient of variation (CV) reflects variability relative to mean force. These indices describe submaximal force consistency rather than peak force-generating capacity. Shoulder external rotation force variability may therefore reveal aspects of submaximal force regulation not captured by maximal strength assessment.

Previous lower-extremity studies have suggested that submaximal force regulation may be related to postural control and dynamic balance performance [9,10]. For example, reduced ankle plantar flexor force steadiness has been associated with greater center-of-pressure fluctuations during single-leg stance tasks [9,10]. In addition, lower-extremity dynamic balance tasks such as the Star Excursion Balance Test are influenced by multiple neuromuscular factors, including lower-limb strength, proprioception, and movement control [11]. These findings provide a conceptual rationale for examining whether force steadiness is related to closed-chain performance tasks. However, whether shoulder external rotation force steadiness is similarly related to closed-chain upper-extremity reach performance remains unclear.

Upper-extremity weight-bearing tasks require coordinated shoulder and trunk control while supporting the body. They may challenge loading capacity, shoulder postural control, trunk control, balance, reach strategy, and force regulation. The upper-extremity Star Excursion Balance Test (UE-SEBT) assesses closed-chain upper-extremity reach performance [10-14]. Participants maintain support through one upper extremity while reaching in multiple directions with the opposite hand. The UE-SEBT thus reflects coordinated upper-extremity, trunk, and whole-body control rather than directly measuring dynamic glenohumeral stability.

Previous studies have reported reduced upper-quarter reach performance in individuals with shoulder impingement syndrome and differences in upper-quarter dynamic balance in overhead athletes with a history of shoulder injury [12,13]. Shoulder external rotation strength deficits have also been reported in patients with shoulder impingement and in overhead athletes at risk of shoulder overuse injury [14,15]. Taken together, these findings suggest that upper-extremity closed-chain performance and shoulder external rotation muscle function may both be affected in populations with shoulder symptoms or high shoulder-loading demands. However, maximal shoulder external rotation strength may not necessarily explain closed-chain reach performance, because such performance likely depends on multiple factors, including force regulation, postural control, trunk control, balance, and reach strategy, rather than maximal force production alone. Therefore, although shoulder external rotation strength and upper-extremity closed-chain performance have each been examined in relation to shoulder disorders, the relationship between shoulder external rotation force steadiness, which may reflect submaximal force regulation of posterior rotator cuff-related external rotation function, and closed-chain upper-extremity reach performance has not been sufficiently clarified.

This exploratory cross-sectional study aimed to examine the associations between shoulder external rotation force steadiness, quantified using the SD and CV of force output during 20% and 40% maximal voluntary force (MVC) contractions, and normalized UE-SEBT reach distances in the medial, superolateral, and inferolateral directions in healthy young adults. MVC peak force was additionally examined as an indicator of maximal force-generating capacity. We expected that lower shoulder external rotation force variability would be associated with greater UE-SEBT reach distances; however, all association analyses were considered exploratory and hypothesis-generating.

## Materials and methods

Participants

Healthy young, non-athletic adults not currently participating in organized or competitive athletic activities were recruited for this exploratory cross-sectional study. Exclusion criteria were current shoulder, elbow, wrist, or hand pain at rest or during upper-extremity movement; previous upper-extremity surgery; or neurological or musculoskeletal conditions affecting upper-extremity performance. Only participants scoring 0 on a visual analog scale for upper-extremity pain were included.

An a priori power analysis for correlation assumed an effect size of r=0.50, an alpha level of 0.05, and a statistical power of 0.80 [16], requiring 26 participants.

The Ethics Review Committee of Kansai University of Welfare Sciences approved the protocol (approval number: 25-18), which followed the Declaration of Helsinki. Participants were recruited and enrolled between December 1, 2025, and March 31, 2026. All provided written informed consent before participation.

Anthropometric measurements

Height, body mass, and upper-limb length were measured before testing. Upper-limb length was measured from the acromion process to the middle fingertip with the shoulder abducted to 90° and the elbow, wrist, and fingers extended. This length was used to normalize UE-SEBT reach distance.

The dominant upper extremity was defined as the arm preferentially used to throw a ball. All measurements were performed by the same investigator.

Shoulder external rotation force testing

Dominant-side shoulder external rotation force was measured using a pull-type handheld dynamometer (HHD; Mobie Z; SakaiMED, Tokyo, Japan) at 100 Hz. The dynamometer was wirelessly connected to a computer displaying real-time force output through the manufacturer’s software (Figure 1). A nonelastic belt fixed the HHD between the distal forearm and a 20-kg dumbbell on the floor [17,18], standardizing resistance and reducing examiner-dependent variation.

**Figure 1:**
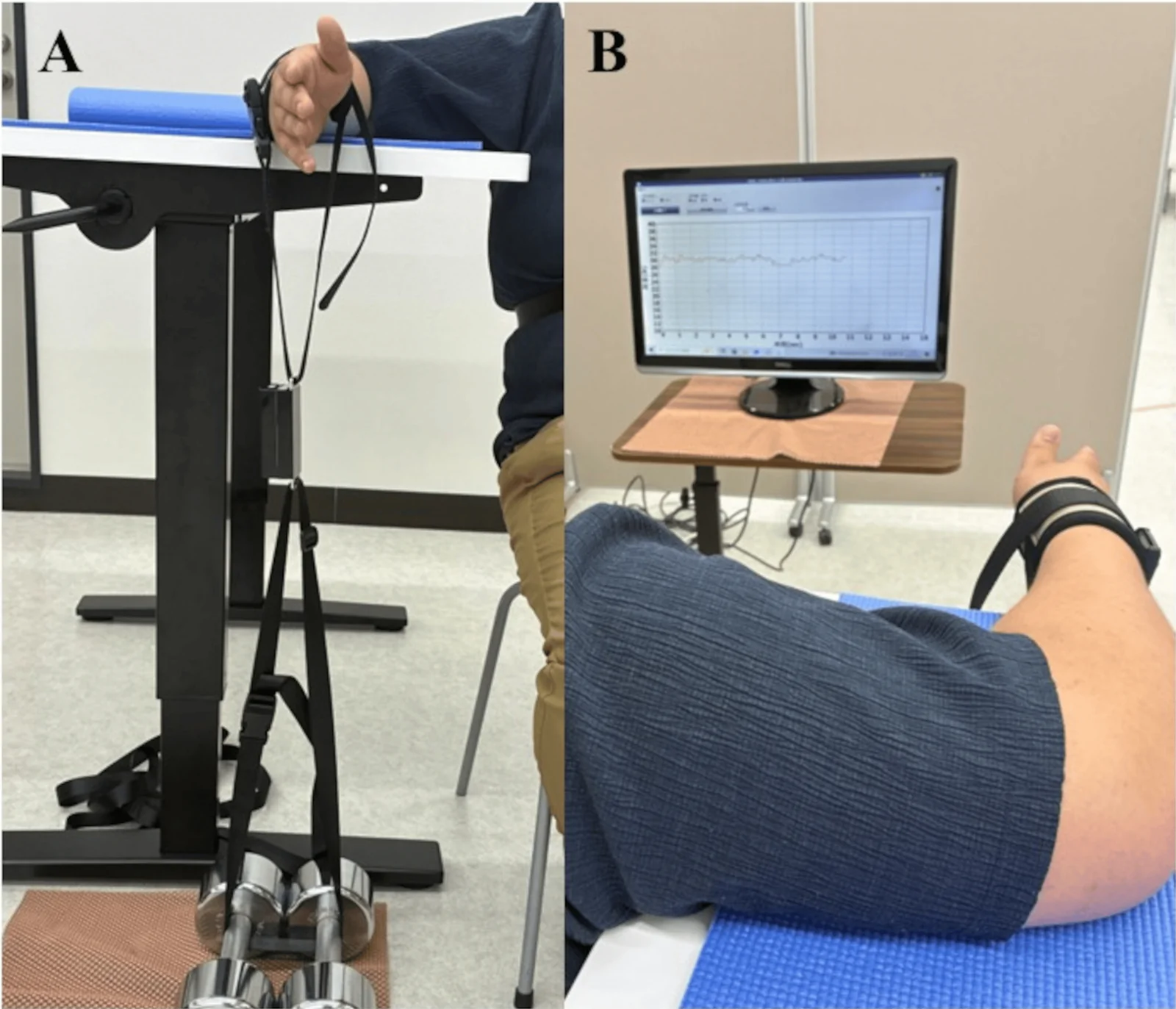
Experimental setup for shoulder external rotation force testing (A) Participant positioning and pull-type handheld dynamometer setup for shoulder external rotation force measurement. The handheld dynamometer was fixed between the participant’s distal forearm and a 20-kg dumbbell placed on the floor using a non-elastic belt. The participant was seated with the shoulder positioned at 90° of abduction and 0° of external rotation, the elbow flexed to 90°, and the forearm in a neutral position. The trunk was partially stabilized with a belt. (B) Real-time visual force feedback display used during the force steadiness task. Participants were instructed to match and maintain the target force as steadily as possible during the 15-second submaximal trials. HHD: Handheld dynamometer.

Participants sat on a chair with a backrest and partial belt stabilization of the trunk. The upper arm and distal forearm rested on a table to maintain position and reduce compensation. The shoulder was positioned at 90° of abduction and 0° of external rotation, the elbow at 90° of flexion, the forearm in neutral pronation-supination, and the wrist at 0° of flexion-extension. Participants were instructed to maintain this posture throughout force testing.

Before formal force testing, participants performed practice trials to familiarize themselves with the shoulder external rotation testing posture, the pull-type HHD setup, and the visually guided force-matching task. The number and duration of practice trials were not recorded.

Maximal Voluntary Isometric Contraction

Participants performed three maximal voluntary isometric shoulder external rotation contractions against the fixed dynamometer, each lasting five seconds. Peak force during each five-second contraction was recorded, with 30-second rests between trials. All participants completed the three MVC trials, and no MVC trials were excluded from the analysis. The three peak forces were averaged for analysis.

Force Steadiness Task

Submaximal force steadiness was assessed at 20% and 40% of each participant’s MVC. These intensities were selected to examine force steadiness under distinct low- and moderate-submaximal load conditions. Previous shoulder external rotation exercise research has examined infraspinatus and posterior deltoid muscle activity using low-, medium-, and high-intensity resistance conditions. Based on this framework, 20%MVC was used as a low-load condition, whereas 40%MVC was used as a moderate-submaximal condition to provide a clear contrast in force demand during the visually guided steadiness task [19]. During the force steadiness task, the participant’s real-time force output and the target force corresponding to either 20% or 40%MVC were simultaneously displayed on a monitor positioned approximately 1 m in front of the participant. Participants were instructed to adjust their force output to match the displayed target and to maintain it as steadily as possible throughout each 15-second trial.

Participants performed three 15-second trials at each intensity, with 30-second rests between trials and a one-minute rest between conditions. The order of the 20%MVC and 40%MVC conditions was randomized.

For force steadiness analysis, a five-second window corresponding to 500 samples at the 100-Hz sampling frequency was used. To avoid the initial and terminal portions of the 15-second trial, the search range was set from one to 14 seconds after trial onset. The five-second window was moved through this search range in one-sample increments, and the window with the lowest SD was extracted from each trial [20]. Mean force, SD, and CV were calculated within this five-second window and averaged across the three trials for the association analyses.

Upper-extremity Star Excursion Balance Test

Closed-chain upper-extremity reach performance was assessed using a three-direction UE-SEBT following previous upper-quarter reach testing procedures. A medial tape line was placed first, with superolateral and inferolateral lines at 135° relative to it [13].

Participants supported themselves through the dominant hand, corresponding to the shoulder external rotation test side. Participants assumed a closed-chain upper-extremity support position with the hands positioned approximately shoulder-width apart, according to previous methods [13]. Participants reached maximally in each direction with the non-supporting hand while maintaining dominant-side support. Before formal UE-SEBT testing, participants performed practice trials to familiarize themselves with the closed-chain support position, reaching directions, and task requirements. The number and duration of practice trials were not recorded.

Excessive pelvic elevation or depression, trunk rotation, and loss of the required support position were prohibited. Before testing, the required starting position and examples of unacceptable compensatory movements were verbally explained and demonstrated to participants. Trials were repeated for loss of supporting-hand contact, failure to return under control, weight-bearing through the reaching hand, or inability to maintain the testing position. Before testing, the required starting position and examples of unacceptable compensatory movements were verbally explained and demonstrated to participants. Reach distance was normalized to upper-limb length [13,21]. Three successful trials per direction were averaged for analysis.

Statistical analysis

All statistical analyses were performed using MATLAB R2025b (MathWorks, Natick, MA, USA). Normality of each variable was assessed using the Shapiro-Wilk test. Descriptive statistics were calculated for all variables.

Associations between HHD-derived shoulder external rotation force variables and UE-SEBT reach distances were examined using correlation analyses. Pearson’s correlation coefficient was used when both variables were normally distributed, and Spearman’s rank correlation coefficient was used when either variable was non-normally distributed.

Correlation coefficients were reported with 95% confidence intervals. Confidence intervals for Pearson’s correlation coefficients were calculated using Fisher’s z transformation. Approximate 95% confidence intervals for Spearman’s rank correlation coefficients were also calculated using Fisher’s z transformation.

Because the association analyses were exploratory and included multiple correlation tests, raw p-values were adjusted using the Benjamini-Hochberg false discovery rate procedure. The false discovery rate was controlled at 0.05 across the zero-order correlation tests between shoulder external rotation force variables and the three UE-SEBT reach directions. Both raw and adjusted p-values were reported, and statistical significance was determined using the adjusted p-values with α=0.05.

To explore the potential influence of body mass on the observed associations, body-mass-adjusted partial correlation analyses were additionally performed for MVC peak force and 20%MVC force variability variables that showed raw p-values below 0.05 in the zero-order correlation analyses. These analyses were considered exploratory sensitivity analyses and were not treated as independent confirmatory analyses.

## Results

Tables 1-3 present participant characteristics and descriptive statistics. Twenty-six healthy young adults (mean age: 20.50±1.00 years) completed the testing. Mean MVC peak force was 116.10±22.20 N. During the 20%MVC task, mean force was 22.55±4.23 N, and 20%MVC SD was 0.69±0.26 N. During the 40%MVC task, mean force was 44.60±8.31 N (Table 2). Mean normalized UE-SEBT distances were 1.34±0.13 medially, 0.80±0.18 superolaterally, and 1.05±0.15 inferolaterally (Table 3).

**Table 1:** Participant characteristics (n=26) Continuous variables are presented as mean and standard deviation. Sex is presented as number of participants. - indicates not applicable.

Variable	Mean or n	Standard deviation
Age, years	20.50	1.00
Height, m	1.65	0.10
Body mass, kg	60.60	15.10
Body mass index, kg/m²	22.10	5.00
Upper-limb length, m	0.70	0.05
Men, n	16	-
Women, n	10	-

**Table 2:** Shoulder external rotation force measures (n = 26) MVC=Maximal voluntary contraction; SD=standard deviation; CV=coefficient of variation.

Variable	Mean	Standard deviation
MVC Peak force, N	116.10	22.20
20%MVC Mean force, N	22.55	4.23
20%MVC SD force N	0.69	0.26
20%MVC CV, %	3.10	1.18
40%MVC Mean force, N	44.60	8.31
40%MVC SD force, N	1.56	0.97
40%MVC CV, %	3.55	2.18

**Table 3:** Normalized upper-extremity Star Excursion Balance Test reach distances (n=26) Reach distances were normalized to upper-limb length. UE-SEBT=upper-extremity Star Excursion Balance Test.

Variable	Mean	Standard deviation
Medial, ratio	1.340	0.129
Superolateral, ratio	0.800	0.177
Inferolateral, ratio	1.045	0.148

Table 4 presents correlations between HHD-derived shoulder external rotation force variables and UE-SEBT reach distances. Analyses included all 26 participants without outlier removal.

**Table 4:** Correlations between shoulder external rotation peak force, force variability, and medial UE-SEBT reach distance (n=26) Values are correlation coefficients with 95% confidence intervals. Raw p values and Benjamini-Hochberg false discovery rate-adjusted p-values are shown separately. MVC=Maximal voluntary contraction; SD=standard deviation; CV=coefficient of variation; UE-SEBT=upper-extremity Star Excursion Balance Test; CI=confidence interval; BH-FDR=Benjamini-Hochberg false discovery rate; Pearson’s r=Pearson’s correlation coefficient; Spearman’s rho=Spearman’s rank correlation coefficient.

Medial	Coefficient	95%CI		p (raw)	p (BH-FDR)
						Lower limit	Upper limit
		MVC peak force, N	-0.177			-0.528	0.226	0.387	0.696
20%MVC SD, N	-0.503	-0.745	-0.144	0.009	0.073
20%MVC CV, %	-0.421	-0.695	-0.040	0.032	0.146
40%MVC SD, N	-0.268	-0.594	0.133	0.186	0.372
40%MVC CV, %	-0.145	-0.504	0.257	0.479	0.767

Correlations between shoulder external rotation force variables and UE-SEBT reach distances are presented separately for the medial, superolateral, and inferolateral directions in Tables 4, 5, and 6, respectively. Before correction for multiple comparisons, 20%MVC SD correlated moderately and negatively with medial reach (ρ=-0.503, 95% CI: -0.745 to -0.144, raw p=0.009) and superolateral reach (ρ=-0.528, 95% CI: -0.760 to -0.176, raw p=0.006). The 20%MVC CV correlated negatively with medial reach (r=-0.421, 95% CI: -0.695 to -0.040, raw p=0.032) and MVC peak force with superolateral reach (r=-0.484, 95% CI: -0.734 to -0.120, raw p=0.012). These four correlation patterns are illustrated in Figure 2.

**Table 5:** Correlations between shoulder external rotation peak force, force variability, and superolateral UE-SEBT reach distance (n = 26) Values are correlation coefficients with 95% confidence intervals. Raw p-values and Benjamini-Hochberg false discovery rate-adjusted p-values are shown separately. MVC=Maximal voluntary contraction; SD=standard deviation; CV=coefficient of variation; UE-SEBT=upper-extremity Star Excursion Balance Test; CI=confidence interval; BH-FDR=Benjamini-Hochberg false discovery rate; Pearson’s r=Pearson’s correlation coefficient; Spearman’s rho=Spearman’s rank correlation coefficient.

Superolateral	Coefficient	95%CI		p (raw)	p (BH-FDR)
						Lower limit	Upper limit
		MVC peak force, N	-0.484			-0.734	-0.120	0.012	0.073
20%MVC SD, N	-0.528	-0.76	-0.176	0.006	0.073
20%MVC CV, %	-0.378	-0.668	0.011	0.057	0.168
40%MVC SD, N	-0.274	-0.598	0.127	0.176	0.372
40%MVC CV, %	-0.071	-0.446	0.326	0.731	0.808

**Table 6:** Correlations between shoulder external rotation peak force, force variability, and inferolateral UE-SEBT reach distance (n=26) Values are correlation coefficients with 95% confidence intervals. Raw p-values and Benjamini-Hochberg false discovery rate-adjusted p values are shown separately. MVC=Maximal voluntary contraction; SD=standard deviation; CV=coefficient of variation; UE-SEBT=upper-extremity Star Excursion Balance Test; CI=confidence interval; BH-FDR=Benjamini-Hochberg false discovery rate; Pearson’s r=Pearson’s correlation coefficient; Spearman’s rho=Spearman’s rank correlation coefficient.

Inferolateral	Coefficient	95%CI		p (raw)	p (BH-FDR)
						Lower limit	Upper limit
		MVC peak force, N	-0.029			-0.412	0.362	0.887	0.900
20%MVC SD, N	-0.026	-0.409	0.365	0.900	0.900
20%MVC CV, %	-0.113	-0.479	0.287	0.582	0.776
40%MVC SD, N	0.064	-0.332	0.440	0.758	0.808
40%MVC CV, %	0.122	-0.278	0.486	0.552	0.776

**Figure 2:**
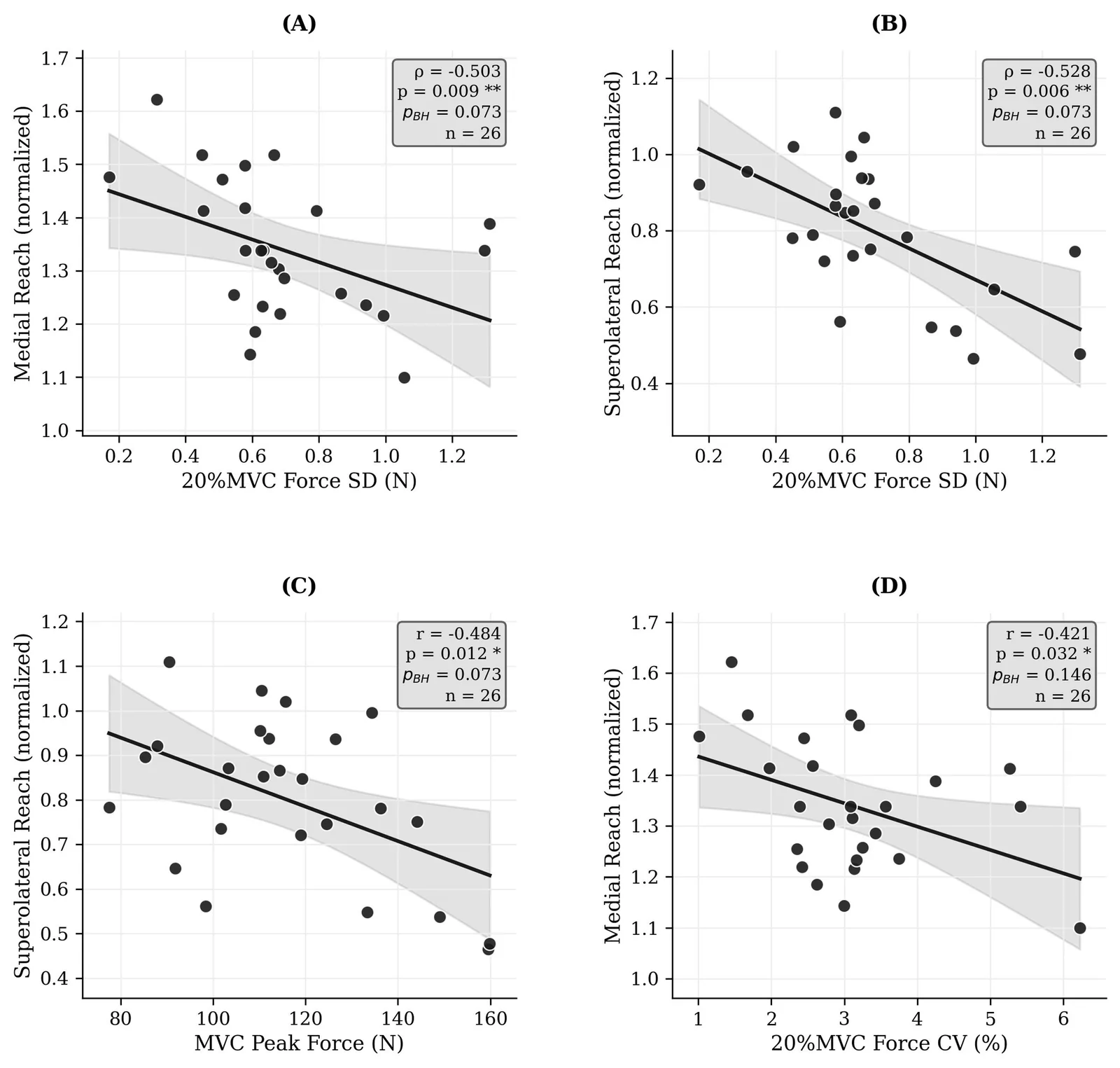
Correlation patterns between shoulder external rotation force variables and upper-extremity Star Excursion Balance Test (UE-SEBT) reach distances in healthy young adults (n=26) Panel A shows the correlation between 20% maximal voluntary contraction force standard deviation and medial reach distance. Panel B shows the correlation between 20% maximal voluntary contraction force standard deviation and superolateral reach distance. Panel C shows the correlation between maximal voluntary contraction peak force and superolateral reach distance. Panel D shows the correlation between 20% maximal voluntary contraction force coefficient of variation and medial reach distance. Reach distances were normalized to upper-limb length. Standard deviation and coefficient of variation were calculated from the five-second window with the lowest standard deviation during the 20% maximal voluntary contraction steadiness task. Spearman’s rho or Pearson’s r is shown according to the normality of the variables. Shaded regions indicate 95% confidence intervals for the fitted line. Asterisks indicate raw p values before correction for multiple comparisons (*p<0.05; **p<0.01). pBH indicates the Benjamini-Hochberg false discovery rate-adjusted p-value. None of the associations remained statistically significant after Benjamini-Hochberg false discovery rate correction. MVC=Maximal voluntary contraction; SD=standard deviation; CV=coefficient of variation; UE-SEBT=upper-extremity Star Excursion Balance Test; BH-FDR=Benjamini-Hochberg false discovery rate.

None remained statistically significant after Benjamini-Hochberg false discovery rate correction. No meaningful correlation patterns were observed for inferolateral reach. Force variability during the 40%MVC task showed no consistent associations with UE-SEBT reach distances.

Exploratory body-mass adjustment attenuated the MVC peak force-superolateral reach correlation (r=-0.484 to partial r=-0.251, p=0.225). Correlations involving 20%MVC force variability changed minimally: 20%MVC SD with medial reach (ρ=-0.503 to partial ρ=-0.518), 20%MVC SD with superolateral reach (ρ=-0.528 to partial ρ=-0.502), and 20%MVC CV with medial reach (r=-0.421 to partial r=-0.420). These sensitivity analyses indicated attenuation of the MVC peak force correlation but minimal change in the 20%MVC force variability correlations.

## Discussion

This exploratory study examined HHD-derived shoulder external rotation force variables and closed-chain upper-extremity reach performance in healthy young adults. Before multiple-comparison correction, lower 20%MVC force variability correlated moderately with greater selected UE-SEBT reach distances: lower 20%MVC SD with medial and superolateral reach, and lower 20%MVC CV with medial reach. MVC peak force correlated negatively with superolateral reach. None remained statistically significant after Benjamini-Hochberg false discovery rate correction. These findings are exploratory and hypothesis-generating rather than confirmatory.

Force steadiness is the ability to maintain a target submaximal force with minimal fluctuation [7,22], distinct from maximal force-generating capacity. SD quantified absolute force variability, whereas CV quantified variability relative to mean force [7,8,23]. The uncorrected correlations suggest that individuals with lower low-load shoulder external rotation force variability tended to reach farther in selected UE-SEBT directions. This interpretation remains preliminary because the associations were not statistically significant after multiple-comparison correction.

These findings are conceptually consistent with lower-extremity studies linking poorer submaximal force regulation to poorer postural control or dynamic balance [9]. Despite different anatomical structures and task demands, lower- and upper-extremity function require force regulation during body support and movement. The findings may extend this concept to upper-extremity closed-chain reach performance, although the associations were exploratory and not statistically significant after multiple-comparison correction.

Correlation patterns mainly involved 20%MVC rather than 40%MVC force variability, suggesting that lower-load force regulation may relate more closely to closed-chain upper-extremity reach performance. The 20%MVC task may require fine force regulation under low force-production demands, whereas the 40%MVC task may involve different control strategies. Lower-load variability may be more relevant to UE-SEBT demands of sustained support and controlled reaching rather than maximal force production. This interpretation remains speculative given the exploratory design, small sample, and absence of electromyography. Future studies should include recreationally active individuals, overhead athletes, and patients with shoulder symptoms. Electromyography could clarify the contributions of the posterior rotator cuff and scapular muscles to lower-intensity shoulder external rotation force regulation.

The moderate negative MVC peak force-superolateral reach correlation before multiple-comparison correction does not demonstrate that greater strength impairs reach performance. It was not statistically significant after false discovery rate correction and weakened after exploratory body-mass adjustment. In contrast, correlations involving 20%MVC force variability changed minimally. Body mass may therefore partly influence the absolute MVC peak force-superolateral reach relationship, whereas low-load force variability may be less body-mass-dependent. This interpretation aligns with lower-extremity studies linking force steadiness to postural control or dynamic balance and suggests information beyond maximal strength. However, it requires confirmation in larger studies.

The UE-SEBT is a multi-segmental closed-chain performance task rather than a direct measure of glenohumeral stability [13,24]. Participants support themselves through one upper extremity while reaching with the opposite hand. Reach distance may therefore depend on upper-extremity loading capacity, shoulder postural control, trunk control, balance, reach strategy, flexibility, and sensorimotor regulation [24-26]. The correlations concern closed-chain reach performance, not direct evidence of shoulder stability or specific rotator cuff function.

These findings primarily inform future research rather than immediate clinical application. In healthy young adults, selected low-load shoulder external rotation force variability indices correlated moderately with UE-SEBT reach distances before multiple-comparison correction. Submaximal force regulation thus warrants investigation as a potential component of closed-chain upper-extremity performance. This study does not establish clinical utility, diagnostic value, or predictive ability. Larger studies should test prospectively defined hypotheses in athletic and clinical populations to examine relevance to rehabilitation, return to sport, or shoulder symptoms.

This study has some limitations. The small sample of healthy young, non-athletic adults limits generalizability to athletes or individuals with shoulder disorders. Although the sample size was determined a priori for detecting a correlation of r=0.50 with 80% statistical power, this calculation was based on a single correlation and was not designed to provide adequate power for the full set of multiple exploratory correlation tests. Therefore, smaller associations may not have been detected reliably, and the present findings require confirmation in larger samples. Analyses were exploratory, with no prospectively specified primary exposure or outcome. Although several raw p-values were below 0.05, no associations remained statistically significant after Benjamini-Hochberg false discovery rate correction. The cross-sectional design prevents causal interpretation. The number and duration of familiarization trials were not recorded; therefore, the potential influence of familiarization on subsequent shoulder external rotation force and UE-SEBT performance could not be quantified. Without electromyography, contributions of the infraspinatus, teres minor, posterior deltoid, and scapular muscles cannot be determined. Selecting the five-second window with the lowest SD may favor the most stable period rather than represent variability throughout the 15-second task. The multi-segmental UE-SEBT cannot directly quantify glenohumeral stability, scapular kinematics, or center-of-pressure control. Finally, despite exploratory body-mass adjustment for selected variables, primary analyses used absolute force values without comprehensively accounting for limb length, body composition, or segmental dimensions.

## Conclusions

In healthy young adults, selected HHD-derived shoulder external rotation force variability indices showed moderate negative correlations with UE-SEBT reach distances before multiple-comparison correction. These associations were not statistically significant after Benjamini-Hochberg false discovery rate correction. Exploratory body-mass adjustment attenuated the MVC peak force correlation but resulted in minimal changes in the selected 20%MVC force variability correlations. Therefore, these findings should be interpreted as exploratory and hypothesis-generating rather than confirmatory evidence of an association. Larger, prospectively designed studies are needed to determine whether shoulder external rotation force variability is associated with closed-chain upper-extremity performance in athletic or clinical populations.
